# Lanthanide Ion Resonance‐Driven Rayleigh Scattering of Nanoparticles for Dual‐Modality Interferometric Scattering Microscopy

**DOI:** 10.1002/advs.202203354

**Published:** 2022-08-17

**Authors:** Lei Ding, Xuchen Shan, Dejiang Wang, Baolei Liu, Ziqing Du, Xiangjun Di, Chaohao Chen, Mahnaz Maddahfar, Ling Zhang, Yuzhi Shi, Peter Reece, Benjamin Halkon, Igor Aharonovich, Xiaoxue Xu, Fan Wang

**Affiliations:** ^1^ School of Mathematical and Physical Sciences Faculty of Science University of Technology Sydney Ultimo New South Wales 2007 Australia; ^2^ School of Electrical and Data Engineering Faculty of Engineering and Information Technology University of Technology Sydney Ultimo New South Wales 2007 Australia; ^3^ School of Physics Beihang University Beijing 100191 China; ^4^ National Key Laboratory of Science and Technology on Micro/Nano Fabrication Department of Micro/Nano Electronics Shanghai Jiao Tong University Shanghai 200240 P. R. China; ^5^ School of Physics The University of New South Wales Kensington New South Wales 2033 Australia; ^6^ Centre for Audio, Acoustics & Vibration Faculty of Engineering & IT University of Technology Sydney Ultimo New South Wales 2007 Australia; ^7^ ARC Centre of Excellence for Transformative Meta‐Optical Systems (TMOS) Faculty of Science University of Technology Sydney Ultimo New South Wales 2007 Australia; ^8^ School of Biomedical Engineering, Faculty of Engineering and Information Technology University of Technology Sydney Ultimo New South Wales 2007 Australia

**Keywords:** interferometric scattering microscopy, lanthanide‐doped nanoparticle, resonance, scattering

## Abstract

Light scattering from nanoparticles is significant in nanoscale imaging, photon confinement. and biosensing. However, engineering the scattering spectrum, traditionally by modifying the geometric feature of particles, requires synthesis and fabrication with nanometre accuracy. Here it is reported that doping lanthanide ions can engineer the scattering properties of low‐refractive‐index nanoparticles. When the excitation wavelength matches the ion resonance frequency of lanthanide ions, the polarizability and the resulted scattering cross‐section of nanoparticles are dramatically enhanced. It is demonstrated that these purposely engineered nanoparticles can be used for interferometric scattering (iSCAT) microscopy. Conceptually, a dual‐modality iSCAT microscopy is further developed to identify different nanoparticle types in living HeLa cells. The work provides insight into engineering the scattering features by doping elements in nanomaterials, further inspiring exploration of the geometry‐independent scattering modulation strategy.

## Graphical abstract

Benefitting from the enhanced resonance of lanthanide ions, the geometry dependence of Rayleigh scattering engineering is broken through by adjusting the ion types in nanoparticles, which results in an efficient and dual‐modality nanoprobe for scattering‐based microscopy.

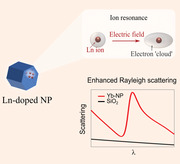

## 1 Introduction

Rayleigh scattering, deviating a light from its straight trajectory, is one of the most important natures of nanoscale objects.^[^ ^1^ ^]^ It reflects the charge distribution and electric polarizability of the nano‐objects under electromagnetic radiation. This intrinsic nature enables the scattering signal to be intense and quenching‐free compared with the fluorescence signal, thus facilitating wide applications, such as detecting bioanalytes,^[^ ^2^ ^]^ tracking proteins^[^ ^3^, ^4^, ^5^, ^6^ ^]^ and viruses,^[^ ^7^ ^]^ building the fluorescent‐free super‐resolution microscopy^[^ ^8^ ^]^ and measuring the time‐resolved carrier dynamics.^[^ ^9^, ^10^, ^11^ ^]^

Scattering spectrum features of nanoparticles are generally governed by the electron interaction. Within metallic materials, according to the Drude model, the excitation beam triggers the oscillation of free electrons and the resultant plasmon resonance. The resonance frequency and amplitude depend on the plasmon resonance mode, electron's effective mass, and concentration.^[^ ^12^ ^]^ In dielectric materials, the electrons are bonded to matrix ions, and the polarization induces a classic Rayleigh scattering where the scattering cross‐section is proportional to the beam wavelength with the power of −4 (≈*λ* ^−4^).^[^ ^1^ ^]^

Controlling the scattering feature from the nanoscale particle is attractive, as the nanoscale size benefits intracellular applications. However, according to the plasmon and dielectric resonance theory, all current methods to modify Rayleigh scattering spectra of nanoparticles are limited to geometry controlling,^[^ ^13^, ^14^, ^15^, ^16^, ^17^, ^18^ ^]^ which often relies on complex fabrication protocols. It is therefore highly desirable to find a robust, geometry‐uncorrelated strategy to harness the scattering spectra of nanoparticles.

The most appealing method to modulate scattering features is achieved by introducing photo‐generated carriers in semiconductor materials. Photon‐generated electron‐hole pairs in a single nanoparticle could modify the complex refractive index and consequently change the scattering spectrum,^[^ ^19^ ^]^ which can be used to inspect the band structure dynamics in semiconductor nanomaterials.^[^ ^10^, ^20^ ^]^ This method has recently been used to track the exciton and charge transport in semiconductors.^[^ ^11^ ^]^ However, the drawback of the method for tunning scattering features is obvious. The modulated scattering signal is considerably smaller than semiconductor nanoparticles’ natural scattered signal due to their high refractive index. Other methods such as differential reflectance spectroscope^[^ ^21^ ^]^ and interference are generally required to extract the modulated scattering feature. Besides, the spectrum modulation range of semiconductors is intrinsically limited to the electronic band structures.

**Figure 1:**
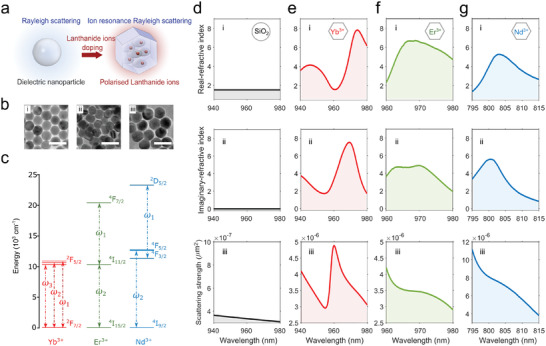
Ytterbium, erbium, and neodymium doping for enhancing the optical scattering. a) Diagram of the resonance effect induced by lanthanide ions doped in NaYF_4_ nanoparticles under the external illumination. b) The transmission electron microscope images of lanthanide‐doped nanocrystals (Ln‐NCs), i) Yb‐NCs, ii) Er‐NCs, and iii) Nd‐NCs, respectively. Here, Yb‐NCs are composed of NaYF_4_: 20%Yb, 2%Er, and the radius is 26.6 nm; Er‐NCs are core–shell–shell structures made up of NaYF_4_: 20%Er@ NaYF_4_: 20%Er@ NaYF_4_: 20%Er and the radius of Er‐NC is 22.5 nm; Nd‐NCs are core–shell–shell structures consisted of NaYF_4_: 60%Yb, 20%Nd@ NaYF_4_: 20%Yb, 2%Er@ NaYF_4_: 40%Nd and the radius of Nd‐NC is 30.9 nm (see Supporting Information for synthesis process). It should be noted that the core–shell structures are employed here for the convenient control of the morphology, size, and doping of Ln‐NCs and have no influence on the resonance effect. The scale bars are 100 nm. c) Energy level diagrams of Yb^3+^, Er^3+^, and Nd^3+^ ions in NaYF_4_ nanocrystal host, where *ω* *is* the dipole resonance angular frequencies. d–g) The numerical modeling of the i) real part and ii) imaginary part of the refractive index, and iii) the scattering cross‐section strength for c) SiO_2_ sphere, d) Yb^3+^, e) Er^3+^, and f) Nd^3+^ doped nanoparticles. The nanoparticles in the simulation are treated as spheres with a radius of 25 nm. The concentration of resonator ions is set as 1.5 nm^−3^ for all three types of Ln‐NCs. The surrounding media is air.

Here we report a new concept of modulating the scattering features of Rayleigh‐regime particles by doping lanthanide ions. Benefiting from the special 4f atomic orbital shielding effect,^[^ ^22^, ^23^ ^]^ the doped ions can maintain their atomic resonance (with host crystal field‐induced Stark levels splitting). The resonance from thousands of lanthanide ions significantly increases the nanoparticles’ Rayleigh scattering strength when the illumination wavelength matches the resonance of lanthanide ions (**Figure** **1**a). Such an efficient biocompatible nanoprobe would benefit scattering‐based microscopies since the scattering feature is immune to shape change and aggregation of nanoparticles. As a proof of concept, we demonstrate the probability of dual‐modality interferometric scattering in living HeLa cells by combining the modalities of fluorescence^[^ ^24^ ^]^ and scattering imaging.

## 2 Results

We first simulate the scattering process for standard SiO_2_ dielectric nanoparticles and lanthanide ions‐doped nanocrystals (Ln‐NCs)^[^ ^25^, ^26^, ^27^ ^]^ in the near‐infrared region. Here we select Ln‐NC as the model to conduct the ion resonance scattering, as the crystalline host NaYF_4_ of Ln‐NC can embed hundreds of thousands of trivalent lanthanide ions, of which the unique 4f electronic configurations are partially filled and shielded by the outer 5s and 5p electrons, providing a rich energy‐level pattern with the adjustable electron‐photon interaction. The NaYF_4_ crystalline host provides precise controlling of doping concentration, doping types, morphology, and size.^[^ ^22^, ^28^, ^29^ ^]^ Other kinds of inorganic hosts (e.g., TiO_2_,^[^ ^30^ ^]^ NaGdF_4_,^[^ ^31^ ^]^ and LiYF_4_^[^ ^32^ ^]^) can also be the doping host.

Under the electromagnetic field of light illumination, these lanthanide ions almost serve as individual dipoles to interact with incident photons in terms of the optical response. In our previous work, we leveraged this optical response to enhance the trap stiffness of optical tweezers.^[^ ^33^ ^]^ Experimentally, the Ln‐NCs can be synthesized with uniform doping concentration and size^[^ ^34^, ^35^, ^36^, ^37^ ^]^ (Figure 1b). The main energy levels of Yb^3+^, Er^3+^, and Nd^3+^ in the NaYF_4_ crystals are calculated according to the absorption curves of nanocrystals at room temperature^[^ ^38^, ^39^, ^40^ ^]^ (Figure 1c). One energy level corresponds to a certain resonance frequency with an energy gap *E*_gap_ = ℏ*ω*. The resonance strength is proportional to the population of carriers that are transitioning between the energy gaps. According to the Rayleigh approximation,^[^ ^41^ ^]^ the scattering cross‐section of a nanoparticle can be expressed as:

(1) $$C_{\mathrm{scat}}\left(\lambda \right)=\frac{8}{3}\pi k_{m}^{4}a^{6}{\left|\frac{\varepsilon_{p}-\varepsilon_{m}}{\varepsilon_{p}+2\cdot \varepsilon_{m}}\right|}^{2}$$

where *k*_m_ = 2*π*/*λ* *n*_m_ is the wavenumber within the surrounding medium; *n*_m_ is the refractive index of the surrounding medium; *a* is the radius of the particle; *ε*_p_ and *ε*_m_ are the permittivity of the particle and the surrounding medium, respectively. The resonance in Stark levels of lanthanide ions will provide extra susceptibility beyond the host material, then the *ε*_p_ for ions with *n* Stark levels can be expressed as:

(2) $$\varepsilon_{p}=\varepsilon_{0}n_{0}^{2}+\sum_{i\;=\;1}^{n}\frac{e^{2}N}{m(\omega_{0_{i}}^{2}-\omega^{2}+j\sigma_{i}\omega )}$$

where *σ*_*i*_ = Δ*ν* · 2*π* is the damping coefficient, *ω* = 2*πc*/*λ* is the excitation angular frequency, $\omega_{0i}$ is the atom resonance angular frequency for level *i*, *ε*_0_ is the permittivity of free space, *m* is the effective mass of an electron, *N* is the number of resonance charges per unit volume, and*n*_0_ is the refractive index of the surrounding medium. Hence the real part and imaginary part of the refractive index of the nanoparticle can be calculated by $n_{p}=\mathrm{real}\left(\sqrt{\varepsilon_{p}}\right)$ and $k_{p}=\mathrm{imag}\left(\sqrt{\varepsilon_{p}}\right)$, respectively. For nanoparticles without nondegenerate energy levels, the *N* is zero, such as SiO_2_ shows a lower refractive index (Figure 1d(i,ii)). Benefiting from the ion resonance effect, both the simulated real part (Figure 1e–g(i)) and imagery part (Figure 1e–g(ii)) of the refractive index from Ln‐NCs indicate a much larger amplitude compared with that for SiO_2_, though the refractive index of the nanocrystal host NaYF_4_ is smaller than that for SiO_2_. The three adjacent Stark levels for Yb^3+^ ions lead to a larger variation with excitation wavelengths. Both Er^3+^ and Nd^3+^ ions have efficient excited‐state absorption with transition angular frequency (*ω*_1_) nearly matching with the ground state's transition angular frequency (*ω*_2_), which in turn results in single peaks in their refractive index response.

Substituting Equation (2) into (1) produces the modified scattering cross‐section for Ln‐NCs. The cross‐section can also be represented by the refractive index as:

(3) $$C_{\mathrm{scat}}\left(\lambda \right)=\frac{8}{3}\pi k_{m}^{4}a^{6}{\left|\frac{n_{p}^{2}-k_{p}^{2}-2\mathrm{ink}-n_{m}^{2}}{n_{p}^{2}-k_{p}^{2}-2\mathrm{ink}+2n_{m}^{2}}\right|}^{2}$$

(4) $$=\frac{8}{3}\pi k_{m}^{4}a^{6}\frac{{\left(n_{p}^{2}+k_{p}^{2}\right)}^{2}-2\left(n_{p}^{2}-k_{p}^{2}\right)n_{m}^{2}+n_{m}^{4}}{{\left(n_{p}^{2}+k_{p}^{2}\right)}^{2}+2\left(n_{p}^{2}-k_{p}^{2}\right)n_{m}^{2}+4n_{m}^{4}}$$

According to Equation (4), interestingly, the scattering would be stronger when the refractive index (both the real and imaginary parts) is larger, and the amplitude would be even larger when the real part is equal to the imaginary part (*n*_p_ = *k*_p_ **)**. The simulated scattering cross‐sections for Ln‐NCs (Figure 1e–g(iii)) show that this effect induces strong fluctuation, especially for the Yb^3+^ doped nanoparticles. The ion resonance‐enhanced scattering cross‐section shows much larger than that of SiO_2_ spheres, around 300 times. Based on Equation (1), the enhancement of the scattering increases with the increasing number of resonance charges per unit volume (*N*), and achieves the maximum when the resonant permittivity of a nanoparticle (*ε*_p_) becomes significantly larger than the permittivity of the surrounding medium (*ε*_m_). The resonance charge number could be increased by doping and pumping more lanthanide ions (e.g., Yb^3+^), as shown in Figure S4, Supporting Information. It is noted that the “*N*” is not the same as the doping concentration of lanthanide ions in nanoparticles. According to the energy transfer theory, the resonance carrier number (*N*) depends on excitation power with the maximum value slightly smaller than the doping concentration since the carriers could release their energy through other pathways such as transferring to Er^3+^ for emission. The excitation power dependent scattering strength of 20% Yb^3+^‐doped nanoparticles is shown in Figure S5, Supporting Information, which shows a similar line shape of Figure S4, Supporting Information. Note that the scattering strength reaches a plateau in Figure S5, Supporting Information is due to excitation saturation.

**Figure 2:**
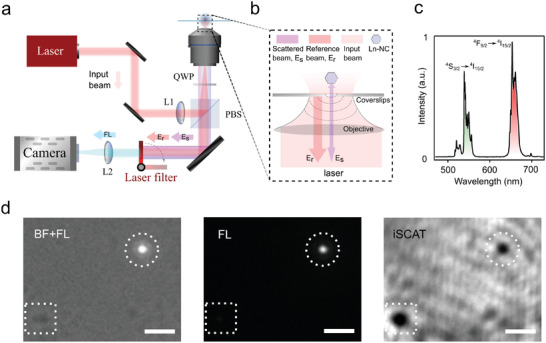
Principle of interferometric scattering (iSCAT) microscopy. a) Sketch of the iSCAT system, where the beam from either Ti:sapphire laser (tunable wavelength from 795 to 815 nm and from 940 to 980 nm for Figure 3) or Thorlabs 980 nm laser (for Figure 4) gets into the oil immersion objective lens (×100, NA = 1.4) via a polarising beam splitter (PBS, 50/50, Thorlabs) and a quarter‐wave plate (QWP). The fluorescence mode is controlled by the flip filter before the camera (see Supporting Information). b) Schematics of iSCAT signal generation. c) The fluorescence spectrum of Yb‐NCs (NaYF_4_: 20%Yb, 2%Er, radius is 26.6 nm) under the illumination wavelength of 980 nm. d) The bright‐filed (BF) image (merged with fluorescence image), fluorescence image (FL), and iSCAT image of Yb‐NCs. The dotted rectangle points to impurities, while the dotted circle represents Yb‐NCs. The scale bars are 2 µm.

To estimate the scattering strength of Ln‐NCs, we employ an interference scattering (iSCAT) microscopy combined with wide‐field fluorescence microscopy. The iSCAT microscopy is a fast, label‐free imaging technology that has been widely explored in tracking nanoparticles of proteins,^[^ ^3^, ^6^, ^42^, ^43^ ^]^ virus,^[^ ^7^, ^44^ ^]^ cell fragments,^[^ ^45^, ^46^, ^47^ ^]^ electrode particle^[^ ^48^ ^]^ and gold nanoparticles,^[^ ^49^ ^]^ and energy flow.^[^ ^11^ ^]^ **Figure** **2**a shows the experimental setup of the iSCAT microscopy, where the input laser is focused on the back aperture of the objective lens. The reflected input beam by the coverslip is the reference beam (*E*_r_ in Figure 2b), and the scattered input beam by the nanoparticle is the scattered beam (*E*_s_ in Figure 2b). Those two beams will overlap at the camera to create the interference image. According to the simulation results, we synthesize three kinds of Ln‐NCs by doping lanthanide ions, Yb^3+^, Er^3+^, and Nd^3+^, in the crystal host of NaYF_4_, respectively (see Supporting Information for synthesis process). In this experiment, we use Yb‐NCs which have the typical emission spectrum (for Yb‐NCs, Yb^3+^ is the sensitizer while the co‐doped Er^3+^ serves as the emitter) as shown in Figure 2c. Figure 2d (right) demonstrates a typical iSCAT image of Yb‐NCs and impurities (e.g., dust particles on the coverglass) that are shown as black dots. Higher contrast of the image (or darker of the black dots) indicates higher scattering strength. We use the in situ fluorescence mode image (Figure 2d middle) to confirm the location of the Yb‐NCs, which is also verified by the merge of bright field and fluorescence images (Figure 2d, left).

**Figure 3:**
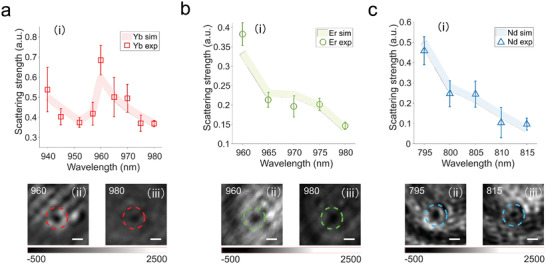
Scattering spectra of Ln‐NCs. The scattering features of Yb‐NCs a‐i), Er‐NCs b‐i) and Nd‐NCs c‐i), respectively. The square, circle, and triangle with error bars represent the experiment data averaged from at least five measurements. The shadow lines suggest the simulated results. Typical iSCAT images of Ln‐NCs under different excitation wavelengths are shown below the corresponding spectrum figures. The Yb‐NCs, Er‐NCs, and Nd‐NCs are labeled by red, green, and blue dotted circles, respectively. The scale bars are 1 µm.

To verify the effect of the ion resonant scattering, we extract the scattering spectra of nanoparticles from a series of iSCAT images with different illumination wavelengths (see Extracting Scattering Amplitude from iSCAT in Supporting Information for extracting method). Applying the scattering extraction method, we obtain the ion resonance‐enhanced Rayleigh scattering for nanoparticles doped with Yb^3+^, Er^3+^, and Nd^3+^ ions, respectively. According to Figure 1, the scattering spectra of Ln‐NCs should have distinct fluctuation with respect to the spectrum of dielectric spheres, as the ion resonance modulates the refractive index. The data points labeled as the square, circle, and triangle in **Figure** **3**a(i)–c(i) are normalized scattering spectra for Yb‐NCs, Er‐NCs, and Nd‐NCs, respectively. These spectra are generally matching with the theoretical simulation (shadow lines in Figure 3a(i)–c(i)), much distinguishable from the scattering feature of low‐refractive index particles such as 300‐nm polystyrene sphere (Figure S6d, Supporting Information). The amplitude mismatches are attributed to the multi‐reflection induced intensity fluctuation. The scattering strength can be directly observed from the visible change in the iSCAT images with a higher signal‐to‐noise ratio representing stronger scattering strength, though the image background intensity varies with wavelengths. The iSCAT image of Yb‐NCs (Figure 3a(ii) black dots labeled by red circles) under 960 nm has higher visibility than that under 980 nm (Figure 3a(iii)), which indicates a higher scattering strength at 960 nm. Similarly, the iSCAT images of Er‐NCs and Nd‐NCs show higher visibility at 960 (Figure 3b(ii)) and 795 nm (Figure 3c(ii)), respectively, than that at 980 (Figure 3b(iii)) and 815 nm (Figure 3c(iii)). Hence Er^3+^ and Nd^3+^ have higher scattering strength at 960 nm and 795 nm. These iSCAT results match the simulation calculated by Equation (3). The measurement confirms that changing the dopant of a nanocrystal could effectively engineer its scattering spectrum, and its iSCAT image can be adjusted by changing the illumination wavelength.

**Figure 4:**
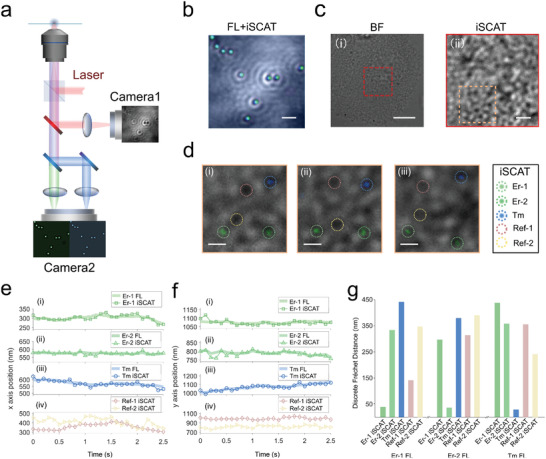
Dual‐modality iSCAT (M‐iSCAT) microscopy for living HeLa cells. a) Schematic diagram of three‐channel M‐iSCAT microscopy. On the typical setup of iSCAT microscopy, we add one dichroic mirror (FF875‐Di01‐25 × 36, Semrock) to separate scattering light and fluorescence light, the two colors of which from two kinds of Ln‐NCs will be further separated by the second dichroic mirror (Di02‐R514‐25 × 36, Semrock). b) Merge of three‐channel images of the 1‐µm polystyrene sphere as position colocalization calibration. In the calibration, the two fluorescence channels are characterized by a wideband white light source through transmission. c) Bright field of a living HeLa cell (left) and the iSCAT image of the red rectangle region in the BF image of the HeLa cell (right). d) M‐iSCAT images of the orange square region at i) 3.4, ii) 3.6, and iii) 4.5 s, in which colorful circles represent different particles as labeled. The Ln‐NCs (Er‐1, Er‐2, and Tm) can be distinguished from reference particles (Ref‐1 and Ref‐2 are intracellular fragments) by merged fluorescence colors. Dependency of fluorescence and iSCAT trajectories of five particles within 2.5 s on e) horizontal and f) vertical axes. g) Dependency valuation of fluorescence and iSCAT trajectories via Fréchet distance, the radial distance of which is calculated taking both x and y positions into consideration. Here Er‐1 and Er‐2 nanoparticles are the Yb,Er‐codoped nanoparticles (NaYF_4_: 20%Yb, 2%Er, radius is 26.6 nm), while Tm nanoparticles are composed of NaYF_4_: 60%Yb, 2%Tm and the radius is 26.5 nm (Figure S1, Supporting Information). The scale bars are b) 2 µm, c, left) 10 µm, c, right) 2 µm, and d) 1 µm, respectively.

This resonance‐enhanced scattering of Ln‐NCs, together with its emission properties, could benefit intracellular iSCAT imaging. Recognition of different types of particles (e.g., distinguishing nanoprobes with respect to vesica) is commonly regarded as a challenge for iSCAT and other scattering‐based methods due to the similar scattering property for particles of similar size. Besides, the abundant cellular structures and fragments significantly increase the difficulties of extracting scattering images of targeting multi‐kind particles in living cells. To address the challenge of distinguishing iSCAT probes while maintaining high imaging speed, taking advantage of the emission of Ln‐NCs, we further demonstrate a dual‐modality iSCAT (M‐iSCAT) imaging by combining the iSCAT channel with two fluorescence channels. **Figure** **4**a depicts the dual‐modality iSCAT microscopy system setup, where Camera 1 and Camera 2 record the conventional iSCAT image and the fluorescence images (FL), respectively. A dual‐viewing optical path splits the fluorescence image into blue light (400 to 514 nm for the Tm‐fluorescence channel) and green light (wavelength between 514 and 785 nm for the Er‐fluorescence channel) on Camera 2. We calibrate the coordinates of iSCAT and two‐color FL images by imaging 1‐µm polystyrene spheres, which results in images overlapping (Figure 4b). Thereby, the FL channels can facilitate locating different types of probes on iSCAT images. The method enables dual‐modality intracellular iSCAT imaging. The intracellular structures (Figure 4c(i)) of living cells often generate many background reference points^[^ ^45^ ^]^ on iSCAT image (Figure 4c(ii)), because of either organelles or localized morphology induced phase change, which challenges the intracellular particle identification and classification. For instance, Ln‐NCs inside a living HeLa cell (Figure 4c(ii)) cannot be distinguished by the conventional iSCAT microscopy. Figure 4d shows the M‐iSCAT images of the squared area in Figure 4c(ii), where the Ln‐NCs (Er‐1, Er‐2, and Tm) can be directly visualized and distinguished from biological particles (e.g., Ref‐1 and Ref‐2) since Tm^3+^ and Er^3+^ doped nanoparticles have blue wavelength band and green wavelength band emission, respectively. The M‐iSCAT images at 3.4, 3.6, and 4.5 s during dynamic tracking (Figure 4d(i–iii)) indicate a recognizable position moving of the Ln‐NCs.

The iSCAT trajectories on both *x* and *y*–axis of Ln‐NCs (Er‐1 iSCAT, Er‐2 iSCAT, and Tm iSCAT) generally match the FL trajectories, as shown in Figure 4e,f. The offset between the iSCAT and FL trajectories would be due to the different imaging frame rates. The iSCAT imaging frame rate is 240 Hz, while the FL imaging rate is 10 Hz, limited by the fluorescence intensity of nanoparticles. Hence, the FL tends to display accumulated positions. Two reference iSCAT points (Ref‐1 and Ref‐2 in Figure 4d) reveal significant different trajectories (Figure 4e(iii),f(iii)) with Ln‐NCs, indicating the dynamics of Ln‐NCs are not from large area structural movement. Hence, the dual‐modality iSCAT could maintain the high frame rate of iSCAT while distinguishing different types of particles. Taking iSCAT images of different laser wavelengths could also be used to distinguish different types of particles due to the different resonance enhancement factors, but it would substantially complicate the systems.

To quantify the correlation between iSCAT and FL trajectories, we calculate the Fréchet distance^[^ ^50^, ^51^ ^]^ as shown in Figure 4g, in which a shorter distance means a stronger correlation. The correlation between Er‐1 FL and Er‐1 iSCAT trajectories shows a Fréchet distance as small as 39 nm, proving that the fluorescence image is from Er‐1 on iSCAT image rather than other points. Similarly, the Fréchet distance for Er‐2 FL and iSCAT trajectories and Tm FL and iSCAT trajectories are 36 and 29 nm, respectively, indicating the fluorescence positions are well connected with the iSCAT positions.

## 3 Conclusion

We demonstrate a method to engineer the scattering spectra of nanoparticles by doping lanthanide ions. We develop the theory to interpret the engineering mechanism and investigate the extraction method of scattering strength from the iSCAT images. Highly doped lanthanide ions benefit the scattering strength of Ln‐NCs under resonant laser illumination by enhancing the susceptibility and the consequent polarizability of the particles. Due to the variation of resonant transition from the types of lanthanide ions, we modulate the scattering features of Ln‐NCs by changing the dopants. The consistency of experimental and simulated results validates the concept of engineering nanoscale objects’ scattering strength.

This strategy provides a novel geometry‐independent path to modulate scattering features, as current methods need to modify the shape or size of particles, such as gold nanoparticles, dielectric particles, and semiconductors. It circumvents the requirement of the second pump laser to excite the particles since the laser serves as both a scattering and excitation source in our system. In this work, we only demonstrate the scattering modulation in the near‐infrared region, however, this resonance theory can be extended to the visible region (see Resonance effect of lanthanide ions (e.g., Er^3+^) in the visible region in Supporting Information). Combining the ion resonance scattering with geometrical resonance engineering, including photonic cavities^[^ ^16^, ^52^ ^]^ and nanoresonator,^[^ ^53^, ^54^, ^55^ ^]^ may further enrich the freedom of scattering modulation. These resonance‐enhanced nanoprobes are not limited to iSCAT microscopy but also work for any scattering‐based methods, such as dark‐field microscopy (Figure S9, Supporting Information). To our knowledge, for the first time, we demonstrated a dual‐modality iSCAT microscopy for intracellular visualizing and classifying nanoparticles in living cells. The dual‐modality iSCAT microscopy takes advantage of the fast frame rate of scattering mode and the particles’ distinguishment ability from the fluorescence mode. Some progress may be achieved if the experimental condition is further optimized, but it is out of our main purpose in this work. For instance, the imaging frame rate of M‐iSCAT can be further improved by using brighter Ln‐NCs and a faster camera, together with advanced data processing method to correlate the FL positions with the iSCAT positions. The imaging channels of M‐iSCAT can be further extended by using different types of Ln‐NCs. In addition, a scattering based (without the need of FL image) multiplexed iSCAT could be achieved by using different wavelengths of scattering laser simultaneously since the scattering efficiencies for different types of nanoparticles are different (Figure 3). We wish this work could open the door to applying a powerful chemical engineering strategy for the modification of Rayleigh scattering and the exploration of new probes for scattering‐based microscopy technologies.

## 4 Experimental Section

The experimental details are provided in the Supporting Information.

## Conflict of Interest

The authors declare no conflict of interest.

## Supporting information

Supporting Information

## Data Availability

The data that support the findings of this study are available from the corresponding author upon reasonable request.
