# Supplementary material for: Lanthanide Ion Resonance‐Driven Rayleigh Scattering of Nanoparticles for Dual‐Modality Interferometric Scattering Microscopy
Source: Adv Sci (Weinh). 2022 Aug 17;9(32):2203354. doi: 10.1002/advs.202203354 (PMC9661846; doi:10.1002/advs.202203354)
Supplement: Supplementary file 1 — Supporting Information [file ADVS-9-2203354-s001.pdf]

ADVANCED SCIENCE
Open Access

Supporting Information

for *Adv. Sci.*, DOI 10.1002/advs.202203354

Lanthanide Ion Resonance-Driven Rayleigh Scattering of Nanoparticles for Dual-Modality Interferometric Scattering Microscopy

*Lei Ding, Xuchen Shan, Dejiang Wang, Baolei Liu**, Ziqing Du, Xiangjun Di, Chaohao Chen*, Mahnaz Maddahfar, Ling Zhang, Yuzhi Shi, Peter Reece, Benjamin Halkon, Igor Aharonovich, Xiaoxue Xu* and Fan Wang**

Supporting Information

Lanthanide ion resonance-driven Rayleigh scattering of nanoparticles for dual-modality interferometric scattering microscopy

Lei Ding, Xuchen Shan, Dejiang Wang, Baolei Liu, Ziqing Du, Xiangjun Di, Chaohao Chen*, Mahnaz Maddahfar, Ling Zhang, Yuzhi Shi, Peter Reece, Benjamin Halkon, Igor Aharonovich, Xiaoxue Xu*, Fan Wang**

Material preparation

Synthesis of lanthanide ions doped nanoparticles. Ln-NCs were synthesized according to the reported method[1,2]. Typically, 1 mmol $RECl_3 \cdot 6H_2O$ (RE = Y, Yb, Er, and Tm) with the desired molar ratio were added to a flask containing 6 mL OA and 15 mL ODE. The mixture was heated to 160 °C under argon flow for 30 min to obtain a clear solution and then cooled down to about 50 °C, followed by the addition of 5 mL methanol solution of NH_4F (4 mmol) and NaOH (2.5 mmol). After stirring for 30 min, the solution was heated to 80 °C under argon flow for 20 min to expel methanol, and then the solution was further heated to 310 °C for another 90 min. Finally, the reaction solution was cooled down to room temperature. The products were precipitated by ethanol and centrifuged (9000 rpm for 5 min), then washed for three times with cyclohexane, ethanol and methanol to get the core nanoparticles. To control the size, nanoparticles grow epitaxially layer-by-layer. The precursors were prepared similar with the method above. The difference is that the precursors solution was obtained after keeping for 20 min at 150°C, instead of further heating to 300 °C to trigger nanocrystal growth. For epitaxial growth, 0.15 mmol as prepared core nanoparticles were added to a three-neck flask containing 6 ml OA and 6 ml ODE. The mixture was heated to 170 °C under argon for 30 min, and then further heated to 300 °C. Next, 0.25 ml as prepared shell precursors were injected into the reaction mixture and ripened at 300 °C for 4 min, followed by the same injection and ripening cycles for several times to get the nanocrystals with the desired size. Finally, the slurry was cooled down to room temperature and the formed nanoparticles were purified according to the same procedure used for the core nanoparticles. In the work, we synthesized four kinds of Ln-NCs, Yb-NCs ($NaYF_4$: 20%Yb, 2%Er, 53 nm), Er-NCs ($NaYF_4$: 20%Er@ $NaYF_4$: 20%Er@ $NaYF_4$: 20%Er, 45 nm), Nd-NCs ($NaYF_4$: 60%Yb, 20%Nd@ $NaYF_4$: 20%Yb, 2%Er@ $NaYF_4$: 40%Nd, 62 nm) and Tm-NCs ($NaYF_4$: 60%Yb, 2%Tm, 53 nm).

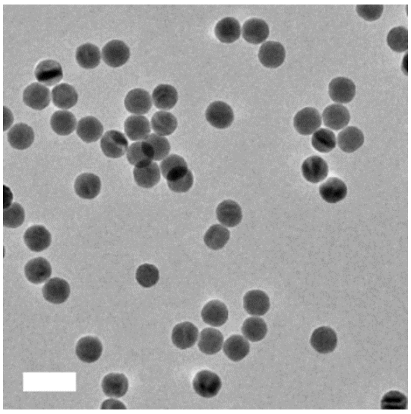

Figure S1. TEM images of the Tm-NCs. The scale bar is 100 nm.

Optical setups

iSCAT microscopy

For Figures 1-3 in the main text, the data was collected by iSCAT microscopy (Figure S2). In this setup, two kinds of lasers were used. One is 980 nm laser diode (BL976-PAG900 - 976 nm, Thorlabs). The other one is a tunable Ti:sapphire laser (Coherent Mira 900 pumped by a Coherent Verdi G). For different lanthanide-doped nanoparticles, we tuned the Ti:sapphire laser every 5nm from 795 to 815 nm for Nd-NCs, from 940 to 980 nm for Yb-NCs, and from 960 to 980 nm for Er-NCs, respectively. During tuning the laser wavelength, we try to keep the similar power for a certain kind of lanthanide-doped nanoparticles, e.g., 167.4±20.0 mW for Nd-NCs, 161.3±13.2 mW for Yb-NCs, and 222±9.7 mW for Er-NCs, respectively. After the polarizing beam splitter, the laser gets through the quarter-wave plate and objective and finally illuminates on the sample. The scattering light, reflected light and fluorescence light are collected by the same objective and finally recorded by a high-speed CMOS camera (Andor Zyla 4.2 plus sCMOS). We use the filter mounted on an adjustable cage to control the iSCAT mode and fluorescence mode.

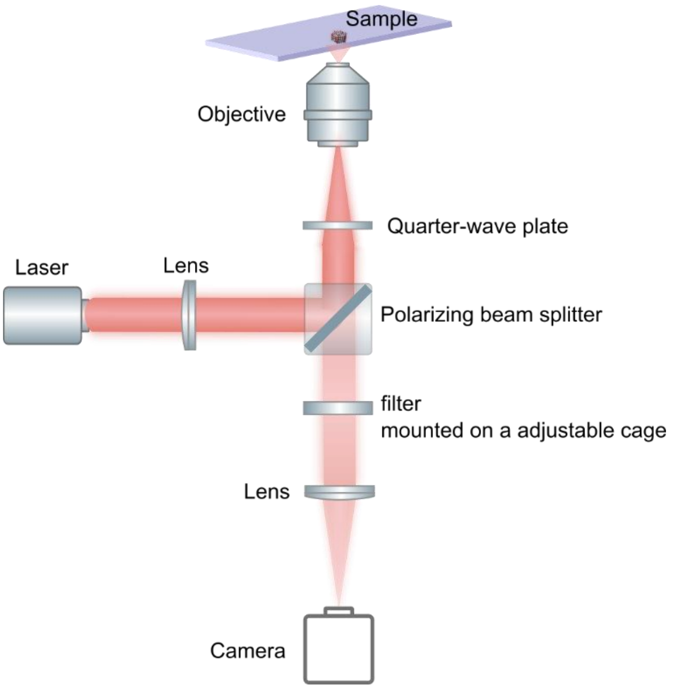

Figure S2. Schematic of the iSCAT microscopy setup.

Dual-modality iSCAT microscopy

For Figure 4, we built a modified iSCAT microscopy by adding separate fluorescence paths to collect blue light and green light. In this setup, the laser is 980 diode from Thorlabs. The camera-1 is a sCMOS (Andor Zyla 4.2 plus sCMOS). The camera-2 is Iris 9 sCMOS Camera (Teledyne Photometrics).

HeLa cells stained by Ln-NCs.

Ln-NCs were modified with POEGMEA$_{13}$-*b*-PMEAP$_7$ di-block copolymers[3]**.** 10 mg Ln-NCs (Er-NCs and Tm-NCs) and 10 mg POEGMEA$_{13}$-*b*-PMEAP$_7$ di-block copolymers (Mw = 6500 g/mol, Figure S3) were dissolved in 1 mL THF and then shaken at room temperature overnight. Next, the Ln-NCs coated with di-block copolymers (Ln-NCs@copolymer) were washed with THF three times and then washed with Milli-Q water twice. Finally, the Ln-NCs@copolymer was dispersed in 500 µL Milli-Q water. Nanoparticles were modified via a ligand exchange process[4].

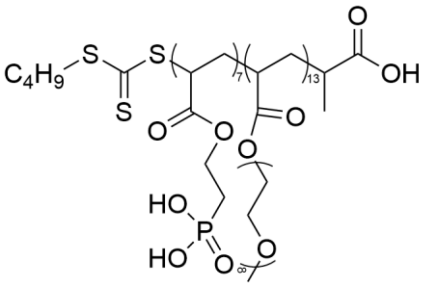

Figure S3. Molecular structure[3] **of POEGMEA$_{13}$-*b*-PMEAP$_7$**.

Cell culture and staining. The HeLa cells were cultured in Dulbecco's Modified Eagle Medium (DMEM) with supplementation of 10% v/v FBS and 1% v/v penicillin–streptomycin at 37°C and seeded on the fluoro-dish (35 mm) culturing for 24 h. For treatment with Ln-NCs@copolymer, the cells were washed with pre-warmed PBS three times and incubated with Ln-NCs (50 µg/ mL, 1 mL) for 1 h. After 1 h, the cells were washed with pre-warmed PBS. Then 2 mL CO_2-independent medium was added to the dish. Finally, the polymer-modified Ln-NCs were taken up by living cells via the endocytosis process and the cell suspension was prepared for iSCAT analysis.

Data acquisition and processing

The iSCAT images in Figures 2-3 were obtained by removing the background of raw iSCAT images. Typically, the reference and signal images of samples were recorded under the

adjustable Ti:sapphire laser or Thorlabs 980 nm laser respectively, and then the processed iSCAT image were generated by dividing reference image with signal image.

The multiplexed iSCAT data in Figure 4 were recorded simultaneously using FL mode and iSCAT mode. For achieving bright enough images, we set the exposure time is 0.1s for FL mode and 0.001s for iSCAT mode. The corresponding frame per second is 10 Hz for FL mode and 240 Hz for iSCAT mode, respectively. The processing method for dynamic iSCAT data was referred to literature[5,6].

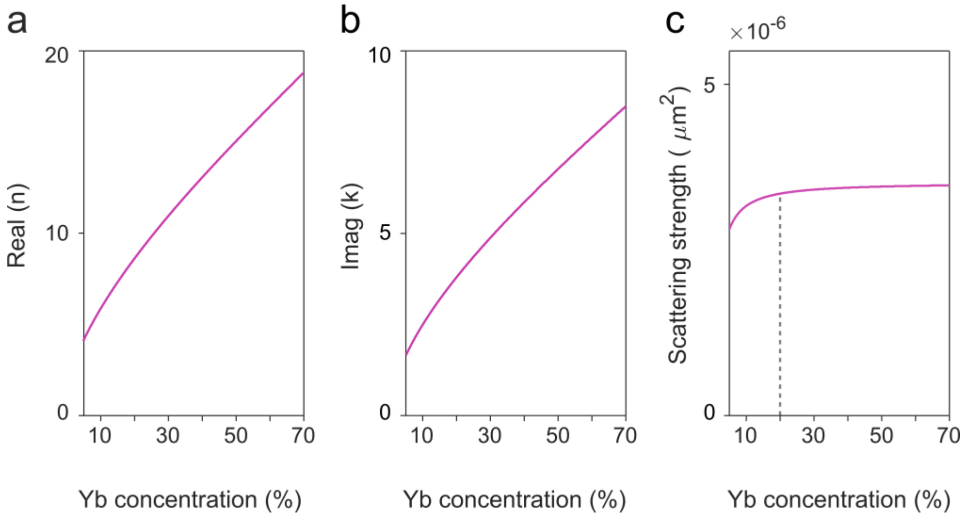

Figure S4. The refractive index and scattering strength vary with the doping concentration of Yb^{3+} (for Yb-NCs). a,b) Both the real part and the imaginary part of the refractive index increase with doping more Yb^{3+} in the host. **c)** Scattering strength of Yb-NCs increases quickly with the doping concentration and gradually achieves the maximum. The power is 50mW, the resonant wavelength is 980 nm, Er^{3+} doping concentration is 2%, and the nanoparticle radius is 25 nm. The dotted line in c represents the result of 20% Yb-doped nanoparticle we used in the main text.

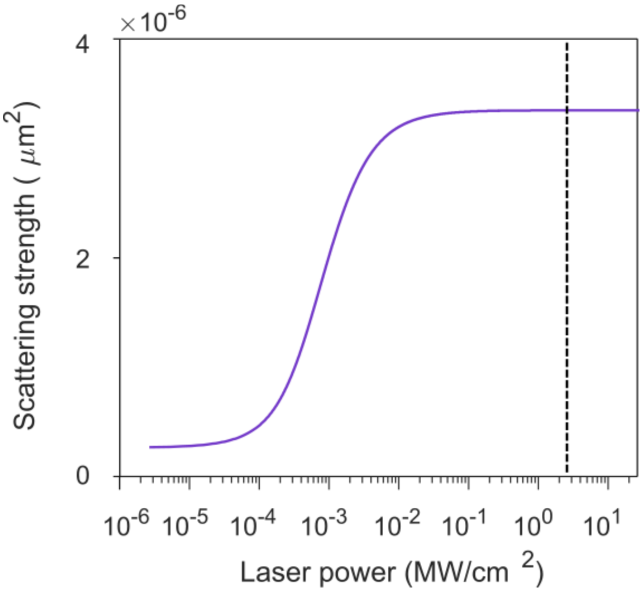

Figure S5. The effect of laser power density on the scattering strength of Yb-doped nanoparticles ($NaYF_4$,20%Yb,2%Er, the radius is 25 nm). The dotted line (black) denotes the power density we used in the simulation in the main text. Comparably, the power density of the spectrometer at 980 nm is around 6×10^{-14} MW/cm^2, where the resonance is close to zero.

Extracting scattering amplitude from iSCAT

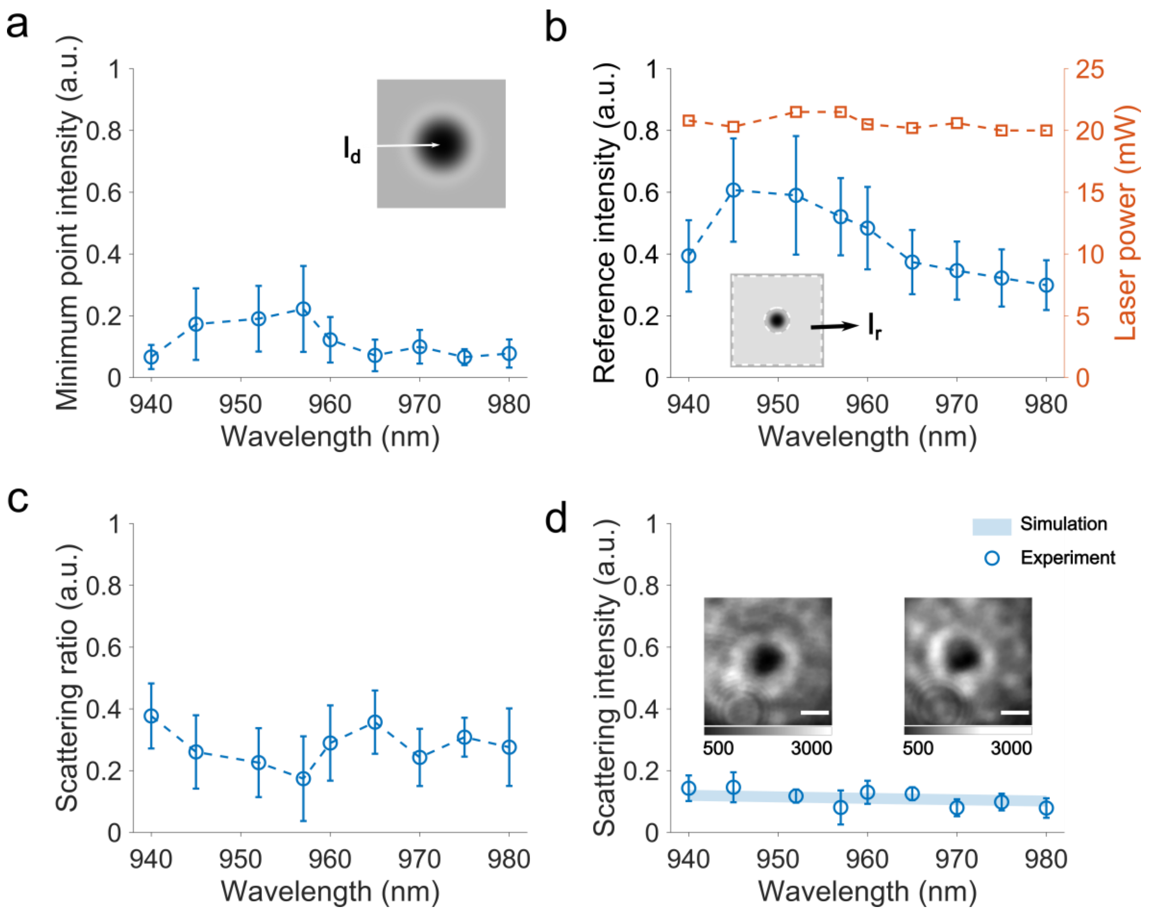

Figure S6. Extracting scattering profile from iSCAT images. a) The normalised detected signal of commercial polystyrene nanosphere with a low refractive index. **b)** The selection of reference light to calculate the scattering spectrum under different illumination wavelengths. **c)** The evaluation of scattering ratio (scattering to reference). **d)** The scattering spectrum of polystyrene nanoparticles. Inset, the background-free images of polystyrene sphere under 940 nm (left) and 980 nm (right) where the background is measured by moving away nanoparticles. The scale bar is 1 μm.

The scattering spectrum of nanoparticles can be extracted from a series of iSCAT images with different illumination wavelengths. The detected intensity (I_d) of the center of nanoparticles in iSCAT is the interference between the reflected beam ($I_r = E_r{}^2$) and the scattered beam ($I_s = E_s{}^2$), expressed as:

$$I_d(\lambda) = I_r(\lambda) + I_s(\lambda) + 2\sqrt{I_r I_s} cos\varphi \tag{1.}$$

where φ is the phase difference between E_r and E_s, consisting of the Gouy phase accumulation, particle-induced phase change and the particle's axial position induced phase change. Figure S6a shows the experimental measured I_d of a 300 nm polystyrene sphere with excitation

wavelength from 940 to 980 nm. Defining the scattering ratio as $\sqrt{I_s/I_r} = C_m$, the equation (1) can be solved as:

$$C_m = \pm\sqrt{cos\varphi^2 - (1 - I_d/I_r)} - cos\varphi \quad (2.)$$

Figure S6b expresses the selection of the $I_r(\lambda)$. In an ideal system that only considers the reflection from the coverslips, the laser power (orange line in Figure S6b) would linearly change with $I_r(\lambda)$. However, in a real-world system, the $I_r(\lambda)$ consists of reflections from multiple surfaces (e.g. lens and PBS), which modifies the intensity for different wavelengths according to the phase change. Hence, we integrate and normalise the intensity of each point on the iSCAT image except the particles' region (Figure S6b, inset), to represent the $I_r(\lambda)$. The sum of intensity (blue line in Figure S6b) shows fluctuation though the input powers are similar for different wavelengths. Here we simplify the $cos\varphi$ to -1, as we make a complete destructive interference by experimental tunning the I_d to the minimum value. This makes the interference most distinct and simultaneously simplifies the data processing. In the case of the scatting signal is smaller than the reflected signal ($I_s < I_r$), the most common cases[7-9] in iSCAT, a negative sign should be used in equation (2), and the solved scattering ratio is shown in Figure S6c. The scattering strength (blue circle in Figure S6d) is calculated by using C_m to multiple I_r, which is matching with the simulated result by Mie scattering theory (blue shadow in Figure S6d). The typical iSCAT images at wavelengths of 940 and 980 nm are shown at the left and right insert figures of Figure S6d, respectively, with similar imaging contrast.

Resonance effect of lanthanide ions (e.g., Er^{3+}) in the visible region.

We extend our resonance theory from the near-infrared region to the visible region. Considering the abundant energy levels of Er^{3+}, we here take the Er^{3+} states from $^4I_{15/2}$ to $^4S_{3/2}$ (Figure S7) to build the rate equation model (equations 1-6). To simplify the situation, we neglect the cross-relaxation possibilities.

Under laser illumination, if the wavelength matches the energy levels, electrons on the ground state of lanthanide ions will be pumped to the excited states (equations 1-5). These excited electrons have different responses with different energy, including nonradiative transition, fluorescence transition and cross-relaxation. Based on these known parameters (Table S1), we solve the rate equation and obtain the active carrier that modulates the refractive index of nanoparticles and produces the resonance effect of scattering strength. Here we analyse the carrier distribution along with the illumination wavelengths from 500 to 530nm and estimate the efficiency of the resonant scattering enhancement (Figure S8). We can see from Figure S8 that the resonance effect appears in the green-wavelength region. Due to the limited resonance carrier number caused by the low quantum efficiency, the refractive index is not obtained substantially enhancement.

$$\frac{dn_1}{dt} = -\frac{\sigma(\lambda)P}{h\upsilon}n_1 + \beta_2 n_2 + \frac{n_2}{\tau_2} + \frac{n_4}{\tau_4} + \frac{n_5}{\tau_5} \tag{1}$$

$$\frac{dn_2}{dt} = -\beta_2 n_2 + \beta_3 n_3 - \frac{n_2}{\tau_2} \tag{2}$$

$$\frac{dn_3}{dt} = -\beta_3 n_3 + \beta_4 n_4 \tag{3}$$

$$\frac{dn_4}{dt} = -\beta_4 n_4 + \beta_5 n_5 - \frac{n_4}{\tau_4} \tag{4}$$

$$\frac{dn_5}{dt} = \frac{\sigma(\lambda)P}{h\upsilon}n_1 - \beta_5 n_5 - \frac{n_5}{\tau_5} \tag{5}$$

$$n_A = n_1 + n_2 + n_3 + n_4 + n_5 \tag{6}$$

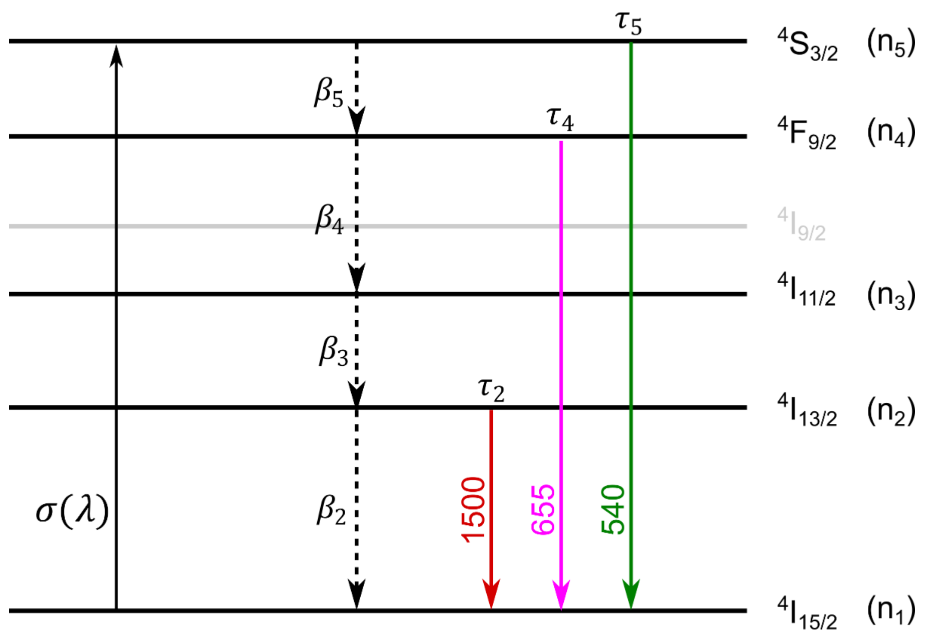

Figure S7. Schematic energy diagrams of Er^{3+}.

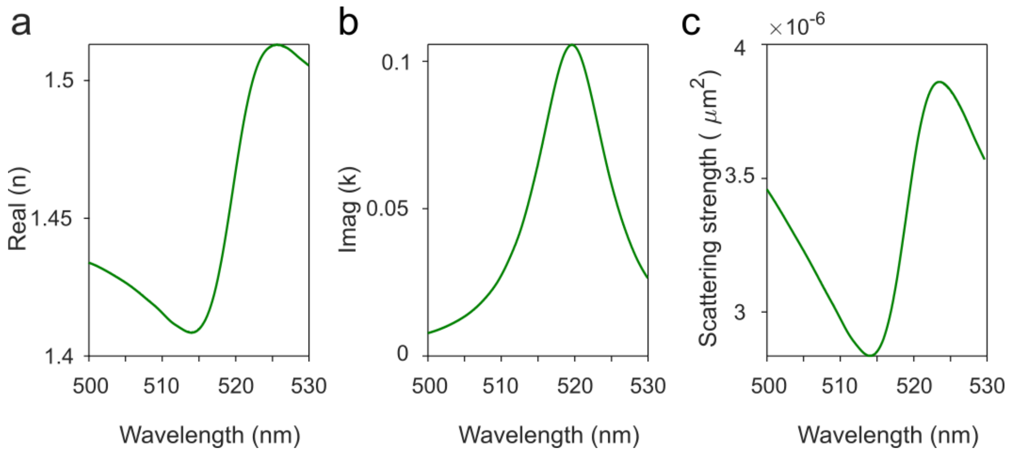

Figure S8 Investigation of the resonance efficiency under illumination wavelength in the visible range. Real part (a) and imaginary part (b) of refractive index, and scattering strength (c) varying with wavelengths. The nanoparticle is Er-NC with a radius of 25 nm. The power is 50 mW.

Table S1. Parameters[10] for Er^{3+}

$\beta_2(s^{-1})$	$\beta_3(s^{-1})$	$\beta_4(s^{-1})$	$\beta_5(s^{-1})$	$P(MW/cm^2)$
8×10^3	3×10^4	4×10^4	5×10^4	13.25
τ_2(ms)	τ_4(ms)	τ_5(ms)	$\sigma(\lambda)$	
500	200	140	Absorption curve	

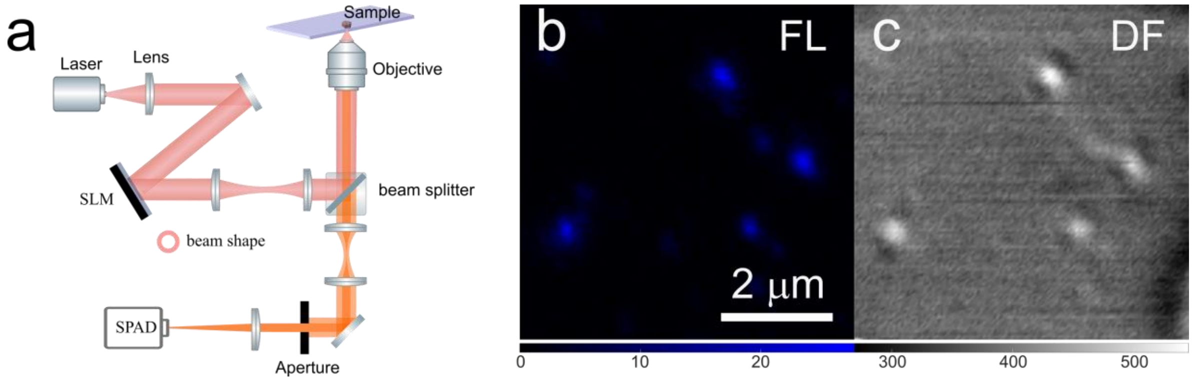

Figure S9. Validation of the nanoprobes in dark-field microscopy. a) Scheme of dark-field microscopy using spatial light modulator as the beam shaper. b) Confocal images of Er-NCs based on fluorescence. c) Confocal dark-field images of the same area with (b) based on scattering. For both modalities, the scanning step is 50 nm, dwell time is 1 ms.

References

1. Liu, Y. *et al.* Amplified stimulated emission in upconversion nanoparticles for superresolution nanoscopy. *Nature* **543**, 229–233 (2017).

2. Duong, H. T. T. *et al.* Systematic investigation of functional ligands for colloidal stable upconversion nanoparticles. *RSC Adv.* **8**, 4842–4849 (2018).

3. Maddahfar, M. *et al.* Stable and highly efficient antibody–nanoparticles conjugation. *Bioconjugate Chem.* **32**, 1146–1155 (2021).

4. Shan, X. *et al.* Optical tweezers beyond refractive index mismatch using highly doped upconversion nanoparticles. *Nat. Nanothechnol.* **16**, 531-537 (2021).

5. Heermann, T. *et al.* Mass-sensitive particle tracking to elucidate the membrane-associated MinDE reaction cycle. *Nat. Methods* **18**, 1239-1246 (2021).

6. Foley, E. *et al.* Mass photometry enables label-free tracking and mass measurement of single proteins on lipid bilayers. *Nat. Methods* **18**, 1247-1252 (2021).

7. Li, N. *et al.* Photonic resonator interferometric scattering microscopy. *Nat. Commun.* **12,** 1744 (2021).

8. Delor, M., Weaver, H. L., Yu, Q. Q. & Ginsberg, N. S. Imaging material functionality through three-dimensional nanoscale tracking of energy flow. *Nat. Mater.* **19**, 56–62 (2020).

9. Young, G. *et al.* Quantitative mass imaging of single biological macromolecules. *Science.* **360**, 423–427 (2018).

10. Guo, X. *et al.* Achieving low-power single-wavelength-pair nanoscopy with NIR-II continuous-wave laser for multi-chromatic probes. *Nat. Commun.* **13**, 1058 (2022).
